# A Systematic Review of Virtual Reality and Robot Therapy as Recent Rehabilitation Technologies Using EEG-Brain–Computer Interface Based on Movement-Related Cortical Potentials

**DOI:** 10.3390/bios12121134

**Published:** 2022-12-06

**Authors:** Ramadhan Rashid Said, Md Belal Bin Heyat, Keer Song, Chao Tian, Zhe Wu

**Affiliations:** 1 School of Life Science and Technology, University of Electronic Science and Technology of China, Chengdu 611731, China; 2 IoT Research Center, College of Computer Science and Software Engineering, Shenzhen University, Shenzhen 518060, China; 3 Franklin College of Arts and Science, University of Georgia, Athens, GA 30602, USA; 4 Department of Women’s Health, Sichuan Cancer Hospital, Chengdu 610044, China

**Keywords:** neurological diseases, electroencephalography, biomedical signal, brain–computer interface, human healthcare, virtual reality, robot therapy, machine learning

## Abstract

To enhance the treatment of motor function impairment, patients’ brain signals for self-control as an external tool may be an extraordinarily hopeful option. For the past 10 years, researchers and clinicians in the brain–computer interface (BCI) field have been using movement-related cortical potential (MRCP) as a control signal in neurorehabilitation applications to induce plasticity by monitoring the intention of action and feedback. Here, we reviewed the research on robot therapy (RT) and virtual reality (VR)-MRCP-based BCI rehabilitation technologies as recent advancements in human healthcare. A list of 18 full-text studies suitable for qualitative review out of 322 articles published between 2000 and 2022 was identified based on inclusion and exclusion criteria. We used PRISMA guidelines for the systematic review, while the PEDro scale was used for quality evaluation. Bibliometric analysis was conducted using the VOSviewer software to identify the relationship and trends of key items. In this review, 4 studies used VR-MRCP, while 14 used RT-MRCP-based BCI neurorehabilitation approaches. The total number of subjects in all identified studies was 107, whereby 4.375 ± 6.3627 were patient subjects and 6.5455 ± 3.0855 were healthy subjects. The type of electrodes, the epoch, classifiers, and the performance information that are being used in the RT- and VR-MRCP-based BCI rehabilitation application are provided in this review. Furthermore, this review also describes the challenges facing this field, solutions, and future directions of these smart human health rehabilitation technologies. By key items relationship and trends analysis, we found that motor control, rehabilitation, and upper limb are important key items in the MRCP-based BCI field. Despite the potential of these rehabilitation technologies, there is a great scarcity of literature related to RT and VR-MRCP-based BCI. However, the information on these rehabilitation methods can be beneficial in developing RT and VR-MRCP-based BCI rehabilitation devices to induce brain plasticity and restore motor impairment. Therefore, this review will provide the basis and references of the MRCP-based BCI used in rehabilitation applications for further clinical and research development.

## 1. Introduction

Neurological disorders lead to a problematic life-compromising medical situation. Globally, neurological disorders are presently the primary cause of motor disability and the second primary cause of mortality, as stated in the current Global Burden of Disease (GBD) study. Their burden has increased over the last 30 years [1,2,3]. Despite advances in traditional motor rehabilitation, persistent motor impairments remain a major issue. Motor function impairment affects patients’ movement, their ability to participate in daily life activities, civic participation, and financial challenges due to their low chances of returning to work. These factors add to the poor overall quality of life [4]. Consequently, forecasting the most effective and efficient rehabilitation treatment interventions for better and prompt functional recovery has received close attention and effort in recent years.

**Figure 1:**
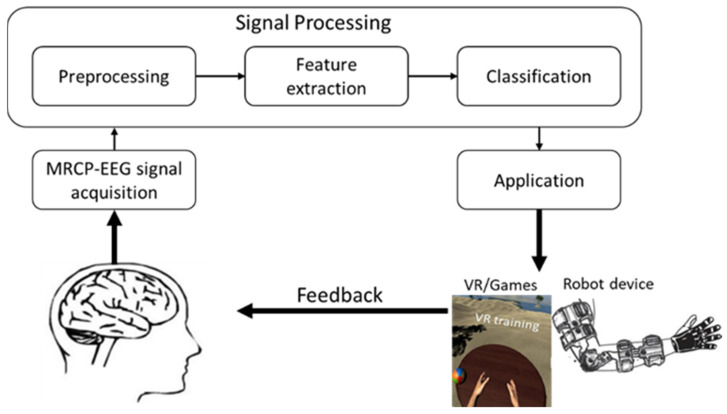
General schematic representation of RT and VR-MRCP-based BCI systems from signal acquisition and signal processing to application in either VR or RT.

Brain–computer interface (BCI) is a computer-based technology that translates brain signals into commands sent to an external function or usage to realize the user’s intention. As a result, patients can communicate with the environment, even though they do not use their peripheral nervous system and muscles [5]. BCI was referred to by a famous Brazilian neuroscientist Miguel Nicolelis [6], as Type 2 hybrid brain–machine interfaces (HBMIs), which control artificial devices through real-time sampling and processing of large-scale brain activity. Currently, brain neural activities are recorded by different BCI techniques and operative methods to extract valuable signals. Noninvasive techniques, such as magnetoencephalography (MEG), electroencephalography (EEG) [7,8,9,10], near-infrared spectroscopy (NIRS), and functional magnetic resonance imaging (fMRI) are prevalently used in human subjects [11]. A BCI system based on EEG has been a more general approach due to its portability, lower cost, flexible adaptation for clinical purposes, and direct measurement of brain neural activities than all previously mentioned BCI approaches [12,13,14]. The electrical brain activity induced by the discharge of electric currents during excitations of the neuron dendrites in synapses is measured by an EEG-based BCI, which is exceedingly sensitive to secondary effects as conductors are positioned on the scalp (Figure 1) [11]. Additionally, EEG-based BCI signals are appropriate for clinical and research purposes because they have high temporal resolution [15]. More details about EEG-based BCIs are found in this literature review [11].

EEG-based BCI employs steady-state visual evoked potentials (SSVEPs), P300 event-related potentials, movement-related cortical potentials (MRCPs), and sensorimotor rhythms (SMRs) as different neuro mechanism types of EEG-based BCIs. Recent research has shown that the Movement-related Cortical Potential (MRCP) extracted from the low-frequency time-domain (LFTD) (0–5 Hz) [16,17,18] approximately 2 s earlier than the onset movement contains enough information to be associated with both intent and executed movements [19]. The magnitude and latency of MRCPs are more modulated by movement features, such as force, speed, and directional information than other methods. MRCPs have been successfully studied for detection using many trials and a single trial [20], making them potentially promising neural control signals. BCI with MRCP as a control signal in neural rehabilitation applications to induce plasticity by monitoring movement intent and feedback is researched [21]. The correlation of MRCP signals in detecting different types of movements and distinguishing between rest and motion has been demonstrated in [16,22], which is why MRCP signals are used in rehabilitation. They reflect many neurophysiological processes [20]. Furthermore, offline or online analyses have done the decoded MRCP signal to control external rehabilitation devices, such as wheelchairs, exoskeletons, and serious games [23]. The MRCP signal is influenced by engagement attention fatigue and the user’s skill level [19,24].

Robot therapy (RT) and virtual reality (VR) MRCP-based BCI systems are two noninvasive rehabilitation technologies that are now being extensively studied to enhance rehabilitation therapy efficacy and functional evaluation for patients with motor impairment. These rehabilitation technologies employ the brain’s neural activity to determine the subject’s movement intention. The most commonly used neural activity is from the motor cortex area of the brain. Various robots and modified commercial games or simple homemade games that mimic the actual movement of the therapist during therapy sessions are currently being used as rehabilitation interventions. Because of the brain neural activity involved in controlling an external device, the potential of employing RT- and VR-MRCP-based BCI systems as rehabilitation methods have drawn much attention. Three fundamental questions will be answered with this systematic review: (i) What type of electrodes and extracted features are used in RT- and VR-MRCP- based BCI rehabilitation systems? (ii) What are the characteristics of MRCP signals in RT- and VR- based BCI? (iii) What are these rehabilitation technologies’ challenges and future potentials?

In rehabilitation, the RT-MRCP-based BCI system approach recognizes the movement intention or execution of a task from the patient using an EEG signal acquisition system [25,26]. The information is decoded and sent to the external robotic device to produce assistive force movement of the paretic limb in a way that imitates the procedures of a therapist in traditional clinical therapy sessions [27,28,29]. The resulting feedback is patient-driven and is designed to bridge the disconnection between movement intent and execution (Figure 1). This method is believed to induce activity-dependent neuroplasticity within brain regions and restore motor function to achieve a specified target [30,31], making the patient’s active participation in rehabilitation exercises an essential element of motor relearning development [32,33]. An RT-MRCP-based BCI system can deliver movement assistance to patients from fully passive to assistive to fully active movements. A combination of RT-MRCP-based BCI therapy and traditional clinical rehabilitation is far more likely to achieve the desired effect of motor function recovery.

**Figure 2:**
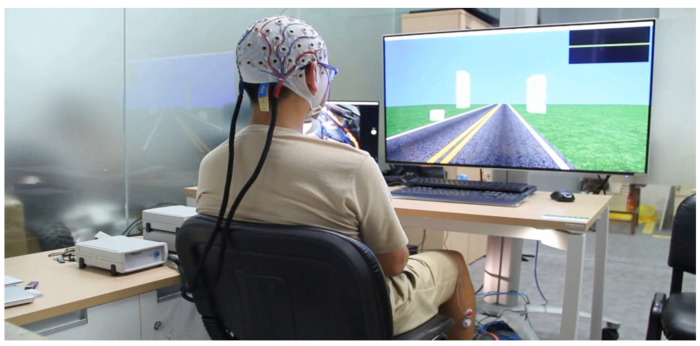
A VR-MRCP-based BCI neurorehabilitation device [47].

VR-MRCP-based BCI therapy as a rehabilitation method in stroke patients stimulates neural networks via the mirror neuron system [34] by utilizing movement visualization, movement intent, and movement imagery [35] that enhance post-stroke motor recovery. One of the fMRI studies revealed that whether or not the virtual limb was shown on the screen, mirror neuron activity could be increased in healthy volunteers during the movement observation task [36]. Thus, it proves a link between VR systems and the mirror neuron system. In applying VR-MRCP-based BCI therapy as a post-stroke rehabilitation method, BCI software records MRCP-EEG signals from the brain as a control signal. Then, the signal is processed, and the required features are extracted. Extracted features are then classified to generate input commands to interact with a virtual environment. The VR software processes the classified input commands to provide meaningful post-stroke patient feedback (Figure 2). MRCP-EEG signals, in conjunction with VR and a BCI, can improve the stimulation of motor brain regions by enhancing the perception of physical movement and the good feeling in VR, thereby involving specific brain neural systems and organizing the required neural plasticity improvements. Various studies have highlighted the advantages of using VR-MRCP-based BCI therapy: VR-MRCP-based BCI therapy provides a safe environment for post-stroke patients to interact with a dynamic and realistic environment [37]. Since motivation has been demonstrated to be important in post-stroke patients, VR-MRCP-based BCI systems can deliver motivation that enhances adherence to the training [38]. Additionally, VR-MRCP-based BCI therapy has various physiological effects on patients by increasing emotional responses [39,40].

Many review articles using various EEG signal modalities have been published on BCI-based EEG neural rehabilitation applications [41,42,43,44,45,46]. However, no research on BCI-based EEG using MRCP as a control signal in neural rehabilitation applications for RT or VR has been published. Thus, reviewing RT and VR-MRCP-based BCI rehabilitation technologies and determining their potential is crucial for designing better treatment interventions for motor impairment. These rehabilitation approaches allow patients with motor dysfunction to regain control of their limbs and build an active neural feedback closed loop to achieve motor function restoration, making full engagement in rehabilitation training visible. Furthermore, these technologies offer promising strategies to modulate neuroplasticity and provide enhanced rehabilitation treatment for patients who have lost limb movement control after a stroke.

This study can support engineers and researchers in RT- and VR-MRCP-based BCI rehabilitation field to improve the existing clinical systems and methods, solve the current challenges and improve the usability of the MRCP-based BCI systems for healthy persons and those with motor impairment. Thus, this review aims to: examine the potential of RT and VR-MRCP-based BCI neurorehabilitation approaches as the adopted methods to translate a patient’s movement intention into an actual movement; bring awareness of this emerging field; and recognize the challenges and prospects in this area of research. The main contributions of this review are as follows:

- A comprehensive survey of RT- and VR-MRCP-based BCI neurorehabilitation approaches using a systematic literature review and bibliographic overlay visualization;
- Identification of the MRCP signal processing approaches, including classifiers and performance measures;
- A review of the MRCP signal preprocessing methods, including epochs, selected electrodes, and applied bandpass filters in RT and VR-MRCP-based BCI neurorehabilitation approaches;
- Provision of the measure for the methodological quality of studies based on the Physiotherapy Evidence Database (PEDro) scale;
- Determining the potential challenges and suggesting solutions for RT- and VR-MRCP-based BCI rehabilitation techniques.

## 2. Methods

We used standard systematic review methods, including search methods, inclusion and exclusion criteria, data collection, and quality assessment, based on Preferred Reporting Items for Systematic Reviews analysis and Metal Analysis (PRISMA) guidelines and the Physiotherapy Evidence Database (PEDro) scale to complete this study. The details of our method are defined below:

### 2.1. Search Methods

**Figure 3:**
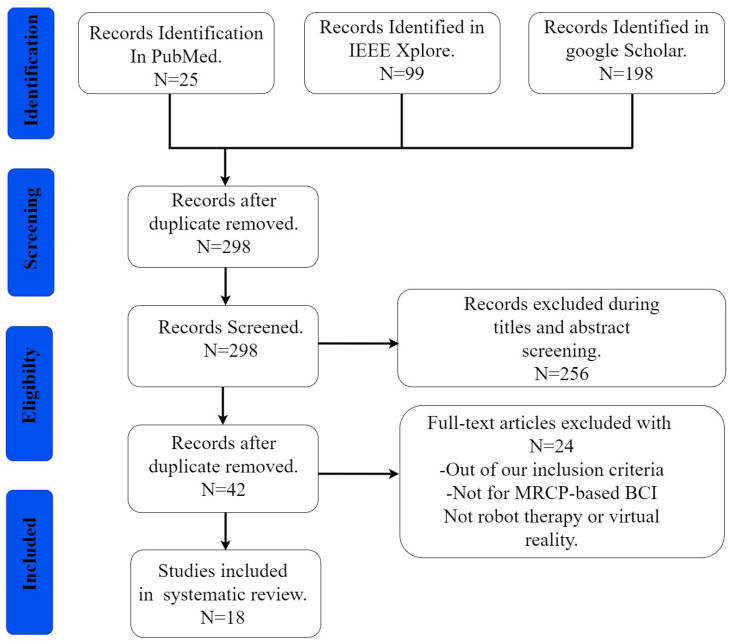
PRISMA flowchart of the present study.

The authors searched major scientific search engines, medical databases, and digital libraries, including Google Scholar, PubMed, and IEEE Xplore databases, for the extensive scientific research articles related to the VR and RT-based BCI rehabilitation technologies for this review. The PRISMA guidelines for systematic review were followed, as presented in Figure 3. The search strategy encompassed studies published between 2000 and March 2022, and it was developed using a combination of standardized Medical Subject Heading terms and keywords (MeSH). These keywords and terms include but are not limited to (Stroke and (acute or subacute) or Seizures or Parkinson’s or Dementia or Epilepsy or limb impairment (upper or lower) and spinal cord injuries) AND (rehabilitation or therapy or treatment or recovery) AND (neural plasticity or neural therapy or motor rehabilitation or therapy or exercise) AND (slow cortical potential or movement-related cortical potential or neuro intention or neuro preparation or neuro anticipation) AND (electroencephalography or brain–computer interface or brain-machine interface or mind–machine interface or neural control interface) AND (virtual reality or serious games or therapeutically games or commercial games) AND (robot or powered exoskeleton or orthosis or assistive arms). The PRISMA checklist is in the Supplementary Materials, and the criteria for inclusion and exclusion are listed in Figure 3.

### 2.2. Inclusion and Exclusion Criteria

The criteria for the inclusion of the searched articles in the review were (i) articles that described the use of MRCP-EEG-based BCIs, (ii) studies that reported on the use of MRCP-BCIs and VR (e.g., serious games, therapeutic games, and rehabilitation games) as treatment interventions, (iii) studies that reported on the use of MRCP-BCI and RT (e.g., exoskeletons and orthoses) and (iv) studies that conducted single or multiple trials on healthy participants or a clinical trial to observe neural reorganization by decoded MRCP signals. The searched articles were also subject to the following exclusion criteria: (i) studies that used other EEG-BCI intention signal modalities (e.g., motor imagery (MI), SSVEP, P300, and sensorimotor rhythms) and (ii) studies that reported the application of BCI RT and VR by using signal modalities other than MRCP-EEG. The full texts of identified articles were then further screened to ensure the articles met the criteria for inclusion in the review. We used the PRISMA literature search checklist to present the final full article results.

### 2.3. Data Extraction

We extracted the following data and result from the full-text articles identified for inclusion in this review: (i) the patient’s or healthy subject’s condition before the rehabilitation intervention; (ii) the applied intervention (RT- or VR-MRCP based BCI); (iii) the MRCP-BCI features, artifact removal, and decoding methods; and (iv) the outcome and performance of the applied method.

### 2.4. Quality Assessment Method

We used the PEDro scale to perform the methodological quality assessment (Table 1). The PEDro scale is based on criteria determined by specialists in physiotherapy practice; it has 11 items found free online [48]. The items included in the PEDro scale are; group comparability at baseline, blinded therapists, eligibility criteria, random assignment of subjects, assignment concealment, blinded subjects, blinded therapists, blind assessors, key results, intention to analyze a key outcome, statistical comparison between groups, and incoherence measures for at least one significant effect. When using the PEDro scale, a higher score indicates better methodological quality.

## 3. Synthesis and Analysis

### 3.1. Key Items Coincidence Analysis

**Figure 4:**
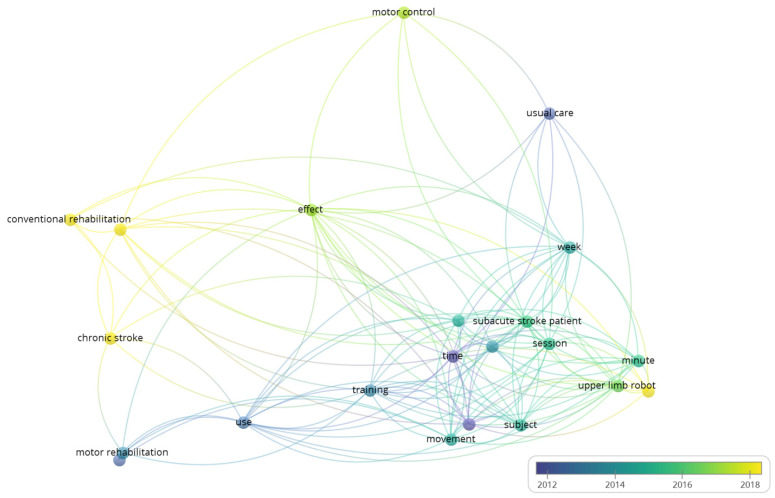
Overlay visualization of key items coincident analysis. The map was constructed by VOSViewer software from the identified 18 studies. The incidences of each key item determined the size of the circle. The colors of the circles indicated the score of the key item since publication according to the color scale.

As part of this review, a map was created based on network data using the VOSviewer tool [65]. This software creates maps, visualizes, and explores them based on any type of network data. In addition, the software is used to identify the relationships between key items as indicators of important systematic research [66,67]. As shown in Figure 4, overlay visualization has been selected as a more effective method of examining the relationships between the chosen key items and time scale elements. The distance between them represents the strength of the two items, the shorter the distance, the stronger the relation. The item that appears more in the publications is shown on the map by a large circle. However, key items were classified by color depending on the year of publication, and yellow circles show the key items found in the most recent publications. As described on the map, 22 key items met the threshold, and the key items, including upper limb, motor control, and rehabilitation classified as the top key items with the most incidence at the average publications above the year 2018, suggesting a new hotspot in MRCP-based BCI rehabilitation field.

### 3.2. Identified Article Results

Initially, the total number of articles discovered was 322; after deduplication, 298 full-text articles remained. Following title and abstract screening, 256 articles were eliminated, leaving only 42 eligible full-text articles. Twenty-four of these articles were later removed after full-text screening for the following reasons: the articles did not meet our inclusion criteria, the design was not for MRCP-BCI, and the review did not involve RT or VR. Finally, a list of 18 full-text studies suitable for qualitative review was identified (Figure 3). In this review, 4 out of the 18 studies used VR-MRCP-based BCI neurorehabilitation approaches, while the rest used RT- MRCP-based BCI neurorehabilitation approaches.

### 3.3. General Information of the Subjects

The total number of subjects from all 18 identified studies is 107, where 4.375 ± 6.3627 are patients and 6.5455 ± 3.0855 are healthy subjects. All studies used either healthy subjects or patients alone or both healthy subjects and patients. The different experimental procedures, few subjects, and varied outcome results make it impossible to analyze potential group differences between healthy subjects and patients. The paper [68] is among the identified articles that enrolled many patients suffering from superior, middle cerebral artery stroke. In contrast, most studies enrolled many healthy subjects [57,59,63]. Several articles performed tests for both healthy subjects and patients. In [64], four SCI patients and three healthy subjects were to test clinical safety and validate the technology; [52] had four healthy subjects and two patients; and [49] had only one patient and two healthy subjects. All of these studies used different procedures for the two groups.

### 3.4. Applied Rehabilitation Methods

In the reviewed articles with RT-MRCP-based BCI neurorehabilitation as therapeutic interventions, the four most frequently used devices were AMADEO, Lokomat, Rex, X1 exoskeleton robot, and BCI-MAFO, shown in Table 2. The lower-limb exoskeleton Rex was primarily used in the studies for gait support without any other external system. Due to its stability, the device can support the subject’s weight and perform crucial tasks such as sitting, walking, standing, and turning by using programmed motions. In [56], the Rex exoskeleton was used to acquire real-world MRCP walking intention data when healthy subjects performed a self-paced movement. The subject-dependent MRCP feature selection method was proposed in [56] on a single trial to detect robust MRCP data. In [57], the Rex exoskeleton was also used to simulate a real-world environment for neurorehabilitation. Healthy subjects used the exoskeleton’s standing and walking forward functions, then MRCP data were collected, and a subject-dependent and section-wise spectral filtering (SSSF) method was used to improve the MRCP data decoding performance.

Furthermore, [54] used the Rex exoskeleton on patients, focusing on walking, turning right/turning left, and sit-rest-stand motion tasks. The MRCP movement intention data were recorded during these tasks, and the offline accuracy was approximately 98%. Zhang [49] also used the Rex exoskeleton to acquire MRCP data from one healthy subject and one spinal cord injury patient. He showed the viability of simultaneously classifying the pattern of the subject’s MRCP movement intention signals and understanding the comparative significance of various scalp brain regions based on the multiple kernel learning (MKL) algorithm. In addition, another lower-limb device was the X1 exoskeleton. The exoskeleton is a wearable robotic device with 10 degrees of freedom (DoF) created in a collaboration between the Institute for Human & Machine Cognition (IHMC) and the National Aeronautics and Space Administration (NASA) ‘s Johnson Space Center. The sequence of elastic actuators with custom motor controllers helps the robot’s motion. The X1 exoskeleton robot alters its joint angles to match the user’s motion, making it relatively easy to use. In [61], the X1 exoskeleton robot was pioneered as a potential assistive, diagnostic, and therapeutic tool for Stroke from the perspective of gait rehabilitation. The author tested the viability of decoding the MRCP-EEG signal of kinetics and kinematics of the lower limb joints during walking with the X1 robot. Other stationary lower-limb robots were the Lokomat, designed on a treadmill [51] and the BCI-MAFO, used while sitting on a chair and designed by Xu [59].

Furthermore, in [15,52,62], the researchers used the customized upper-limb exoskeleton AMADEO robotic device manufactured by Tyro motion GmbH, Bahnhofgürtel 59, 8020 Graz, Austria. AMADEO is a distal upper-limb motor recovery end-effector robot-assistive device for post-stroke patients. AMADEO enables active and passive fingers to produce different finger and thumb motion patterns. The AMADEO robot device’s joints have 5 DoF and present four modes of instruction, including passive training, passive training with biofeedback, assistive training together, and active 2D training games [62]. In [52], four healthy subjects and two post-stroke patients used the AMADEO robotic device to detect intention signals during the visual cues and AMADEO game training protocols. The effectiveness of the two training protocols was more evident during the AMADEO game training protocol for healthy subjects and patients.

Similarly, [15] trained one stroke patient on the AMADEO robot to detect the MRCP amplitude signal at different training levels. The results indicated that the subject recovered the most when fully participating in the exercise. In addition, [62] conducted two-stage (4 and 8 weeks) AMADEO hand motor-assisted rehabilitation robotic training in three post-stroke patients to investigate whether one or combined rehabilitation phases significantly impact motor recovery and neuroplasticity. The results demonstrated that using both training stages effectively improved neuroplasticity changes and hand-motor abilities but using only one training stage did not. The four studies identified used different BCI designs RT capable of movement training during rehabilitation [53,55,60,64]. Another identified therapeutic intervention from this review was VR-MRCP-based BCI neurorehabilitation, which provides exciting visual feedback for activating and monitoring the effect of targeted neurons. The targeted brain neurons determined the type and intensity of neurorehabilitation [69]. Thus, as indicated in [70], VR-MRCP-based BCI systems enhance traditional rehabilitation therapy by promoting self-assisted training, motivation, and good performance with a pleasant treatment experience, including real-time movement neurofeedback.

We identified four studies that employed VR-MRCP-based BCI therapy [47,50,58,63]. In [50], the authors demonstrated how to control virtual avatar walking movements by decoding the EEG signal of the lower-limb angle of joints from the scalp in a real-time closed-loop BCI during treadmill walking. This review recruited two healthy male subjects to walk on a treadmill. The designed neural decoder decoded brain neural activity in real-time to lower-limb movements through scalp EEG. It was observed that the subjects managed to control the avatar gait patterns in the virtual environment through MRCP-BCI signals that were generated from the subject’s scalp. The more subjects controlled the walking avatar, the more walking improved, suggesting that the virtual avatar triggers cortical plasticity.

Additionally, [63] introduced an electro-tactile menu and a brain switch for online closed-loop control using a BCI. The healthy subjects successfully achieved high achievement on a target-hitting task by moving a 2D cursor in a specified direction and stopping. Furthermore, to investigate brain neural activity behavior during unusual real-life situations, [47] demonstrated how MRCPs behave during non-emergencies and emergencies. They designed two VR BCI games to match emergency and non-emergency environments. Seven healthy subjects were recruited to participate in all the experiments. There was a significant difference between the two tasks. The accuracy in the non-emergency task was higher than that in the emergency task. This finding suggests that the environment must be considered when a VR-MRCP-based BCI is applied to brain neural activity rehabilitation. Thus, a set of rules recognized in a normal environment may not be suitable during an emergency.

### 3.5. Signal Acquisition and Processing

The MRCP signal is the typical signal associated with movement intention or execution. In 17 out of 18 identified studies, MRCP signals were generated in the Bereitschaft potential (asynchronous), which is known as a self-paced method. Only [61] showed the MRCP signal generated in a contingent negative variation (synchronized), known as a cue-based method. Offline analysis of MRCP signals was mostly used to process the signals in the identified studies; only five studies used online analysis to process the MRCP signals. As proposed in [71], offline processing produces precise and valuable results, while online analysis may produce lower-quality results. However, in [72], the offline and online signals were explored, and the results were compared. Higher accuracy was obtained from each subject in online BCI models than in offline BCI models, suggesting that BCI exoskeleton online analysis applications are preferred. All reviewed studies in this work showed that the supplementary motor area (SMA) and cingulate motor areas are particularly evolved in pre-movement preparation and readiness for voluntary movement.

As shown in Table 3, all of the identified studies that were reviewed used electrodes on various scalp locations to obtain the MRCP signal. Before further processing, the MRCP signal must undergo several preprocessing procedures, such as filtering and artifact removal. In identified studies, the frequency range of the MRCP signal was bandpass filtered primarily between 0.01 and 5 Hz using different signal processing filters that decrease and flatten out high-frequency noise related to a measurement. Furthermore, the Cz C1, C2, and C3 channels are most commonly used to detect motor intention using the MRCP signal, with C3 being demonstrated to be for right-hand motor intention.

### 3.6. Performance of the Applied Method

The identified studies did not clearly explain evidence of the performance of the applied intervention, which may be because most studies tested the intervention to determine its methodology, and no clinical outcome evaluations were carried out to validate the methods (Table 3)**.** The low number of healthy subjects and patients and the differences in the experimental procedures and performance measures applied make between-study comparisons unfeasible. In theory, studies can be compared using the same frame of reference (i.e., number of subjects, procedures, and performance measures).

However, to evaluate the performance of the applied intervention methods, the identified studies used various performance measures with different detection algorithms. Sensitivity and accuracy were the measures used for assessing performance in the identified studies, as shown in Table 3. *Sensitivity* and *accuracy* are calculated using Equations (1) and (2), respectively, where *TP*, *TN*, *FP*, and *FN* represent the true-positive, true-negative, false-positive, and false-negative numbers, respectively [73,74].

(1) $$\;Sensitivity=\frac{TP}{TP+FN}$$

(2) $$Accuracy=\frac{TP+TN}{TP+TN+FN+FP}$$

The likelihood that a model accurately predicts recognized movements, or the true-positive rate (TPR), is known as sensitivity. The hypothesis in this situation of manipulating an external device is that the individual intends to move. Five studies used sensitivity measures to measure their model’s performance [47,55,58,59,64]. In [55], seven healthy subjects were recruited to test a proposed new concept of communication and control based on the BCI system. The intention of distinguishing presented choices and selecting the desired choice was detected online by scalp MRCP-EEG using locality-preserving projection followed by linear discriminant analysis (LPP-LDA). The concept’s viability was demonstrated with a four-choice BCI, which generated 80% and 70% sensitivity for the movement and intention selection commands. Similarly, the LPP-LDA classifier was used in [59] to detect MRCP movement intention. Ten healthy subjects were required to perform self-paced ballistic dorsiflexion movements of their right foot using motorized ankle-foot orthotics. The sensitivity achieved was 73.0 ± 10.3% in the online analysis.

Furthermore, when the number of features was large in comparison to the number of observations, a sparse discriminant analysis (SDA) classifier was used to investigate the feasibility of using a closed-loop BMI to control an ambulatory exoskeleton with no balance support. The MRCP signals of three healthy subjects and four spinal cord injury patients were used to decrypt their movement intention and activate exoskeleton movement. The results showed that the sensitivity for healthy participants was 84.44 ± 14.56%, while the sensitivity for spinal cord injury patients was 77.61 ± 14.72% [64]. Furthermore, to maximize the signal-to-noise ratio, a matched filter was used with a support vector machine (SVM) [47] to detect the differences between MRCP during an emergency and non-emergency state in VR environment tasks. The sensitivity attained was 60.57 ± 14.79% in non-emergency states and 44.29 ± 5.73% in emergency states. As with other performance measures, accuracy can be defined as the ratio between correctly observed and wrongly observed movements to the total number of observed movements. Four studies among the identified studies used accuracy, in percentages, as the performance measure [50,52,57,63,75].

Two other studies defined *accuracy* as the percentage of EEG samples correctly classified [49,54]. In [52], SVM was used to detect MRCP-(EEG-BCI) signals from different movement training using robot assistance. The classification accuracy achieved was 79.7% for healthy subjects and 66.64% for post-stroke subjects. In [49], the MKL algorithm was used to classify gait states from EEG signals. The results from the tested subjects achieved average accuracies of over 90% for the two types of exercise classification tasks and over 65% for the other four complex types of exercise classification tasks. Regarding singularity challenges, regularized LDA (RLDA) offers a useful answer. In [56], Jeong 2017 used RLDA to decode MRCP walking intention under the lower-limb exoskeleton. Grand average classification accuracy of 87.6% was attained by five healthy participants who performed a self-initiated walking task while wearing the exoskeleton. In [54], he proposed the use of a local Fisher’s discriminant analysis (LFDA) followed by a Gaussian mixture model (GMM) classifier to decode subjects’ motion intentions while wearing a lower-body exoskeleton. The offline assessment accuracies were approximately 98%. Jeong 2020 [57] proposed SSSF as a powerful machine learning filtering method to accurately determine a user’s intention on a single trial. Decoding results using RLDA reached 86%, and public dataset performance was 73% for all subjects. Six studies did not present any performance results [15,51,53,60,61,62].

MRCP-BCI has been highly explored in the past 10 years because it is associated with movement intention and execution [16,59,76,77,78]. Many selected EEG electrodes were used in the most identified studies, and the MRCP-EEG signal was filtered in a lower frequency-time domain. A combination of LPP-LDA and LFDA-GMM classification methods applied in [54,55,59,63] were observed to perform better at minimum latency. Hybridized classifiers have been demonstrated to minimize large dimensionality problems and to improve prediction accuracy [79,80,81]. However, SVM, LDA, and SDA had better performance among the simple classifiers, as depicted in Table 3, because they have a low variance and a high bias. In contrast, complex classifiers have the opposite [82]. Usually, the number of datasets determines which classifier to use to achieve better accuracy. The dimensionality is proposed to be at least five to ten times the number of training samples per class [83,84].

Although the mode of MRCP-BCI may affect the efficiency of the system, both self-paced (online or offline) and cue-based (offline or online) methods have been shown to induce neural plasticity, as mentioned in several studies [85,86,87]. In most identified studies, self-paced offline analysis was used, as indicated in Table 4. This is because the self-paced offline analysis can accurately detect changes in neural activity and promotes engagement with rehabilitation sessions compared to cue-based (offline or online) systems. Furthermore, MRCP-BCI was accurately detected with low latency, allowing enough time for an external device to provide relevant sensorimotor feedback and reach the cortical level [68,88].

## 4. Discussion

This systematic review mainly focused on the potential of closed-loop RT- and VR-MRCP-based BCI neurorehabilitation methods to translate chronic stroke patients’ movement intentions into actual movements. Eighteen studies published between 2000 and 2021 were collected for this review. This chapter discusses a summary of each MRCP-BCI rehabilitation system and its challenges. Finally, the reliability of the rehabilitation methods is analyzed, along with the potential future rehabilitation methods for post-stroke patients.

One of the outcome rehabilitation methods from this review is closed-loop RT-MRCP-based BCI neurorehabilitation. The MRCP movement intention signal is decoded from the brain activity, and the external device initiates the required motion related to the user’s intention to perform specific movements. The advantage of these rehabilitation methods is that they can be used even in patients with no motor function and help them to interact with their external environment. Various studies have shown that this rehabilitation method is promising for inducing plasticity. It facilitates neural activity training with continual repetition and simultaneously offers motion support.

Nevertheless, because the MRCP signal is very weak and is heavily influenced by the subject’s attention and fatigue, maintaining the robot’s movement in real-time settings is a significant problem because post-stroke patients quickly become tired and find it difficult to concentrate on a task. Moreover, another limitation of RT-MRCP-based BCI neurorehabilitation is that its operating system is cumbersome, complex, and massive. The patients may lose motivation and feel uneasy in performing training exercises.

Another evaluation of rehabilitation methodology was closed-loop VR-MRCP-based BCI neurorehabilitation, which was developed to provide rewards and encourage patients to fully engage in the rehabilitation training program, thereby overcoming the problem of patients becoming bored and dropping out. VR-MRCP-based BCI neurorehabilitation is more cost-effective than RT-MRCP-based BCI neurorehabilitation. It directly interacts with the physical environment, boosting user concentration to participate in the task and activating more brain neural networks to induce plasticity. VR-MRCP-based BCI neurorehabilitation can be used with or without a therapist present. Despite these enormous advantages, few studies have been published, perhaps because VR-MRCP-based BCI neurorehabilitation is a new rehabilitation method. A small sample of healthy subjects and post-stroke patients have been involved in testing the implementation of the techniques. Additionally, post-stroke patients find it difficult to interact with commercial VR games due to the functional complexity of the games, which were customized for healthy subjects. Therefore, customized VR neurorehabilitation should be designed to match post-stroke patients’ needs in a realistic environment. Hence, further in-depth exploration should be conducted to apply the method and study the viability of VR-MRCP-based BCI neurorehabilitation approaches in post-stroke patients.

Regarding Table 4, small samples of healthy participants were evident in most reviewed studies. Nevertheless, some commercial RT-MRCP-based BCI systems, such as AMADEO, Lokomat, Rex exoskeleton, XI robot, and BCI-MAFO, were alleged to be as valuable and practical as VR-MRCP-based BCI systems, as in [47,50], and were shown to be helpful in healthy subjects. Still, the effectiveness of these rehabilitation approaches in a large number of post-stroke patients is debatable. Hence, more clinical trials should be conducted to support these claims. Moreover, another remaining problem is to turn these complex rehabilitation methods into cost-effective, user-friendly, and small systems that can be used regularly at home.

Home-based neurorehabilitation devices would improve the quality of life of post-stroke patients. They would allow low-cost therapy to be performed without therapist supervision or the need to schedule rehabilitation sessions. Another potential concern is the scalp’s lack of a standardized electrode location to record MRCP signals. MRCP signals were decoded using electrodes placed at various locations on the scalp in the studies mentioned in this review. In this review, the Cz and C3 channels are the most commonly used to detect motor intention using the MRCP signal, with C3 being demonstrated to be for right-hand motor intention. However, only [52] found FC3 to be the most consistent electrode site for detecting hand motor intention. In another work [89], fMRI was used to study the Bereitschafts-BOLD response, and it showed that the supplementary motor area (SMA) and cingulate motor areas are particularly evolved in pre-movement preparation and readiness for voluntary movement. MRCP signals were also observed in the motor cortex area [90].

Furthermore, hybrid LPP-LDA classifiers were mainly used and attained high performance in many studies [55,59,63], even though careful studies must still be conducted to minimize noise and other artifacts. The importance of generalization skills is sometimes neglected. Improvements in skill generalization might be a solution to the classifiers. The number of training repetitions; the force used; and difficulties in the training, attention, and pathological lesions in specific brain areas influence the amplitude and latency of the MRCP signal [16]. The classifier is an independent factor during preprocessing of the MRCP signal, whereas the frequency and the spatial filters are dependent factors [91]. As a result, robust detection with low latency and high accuracy is required for an RT- or VR-MRCP-based BCI neurorehabilitation system to be useful.

### Limitation

However, there are several limitations to this review. First, a group comparison analysis of the two rehabilitation methods was not performed because of the small number of studies obtained from only three databases with restricted publication years. For this reason, meta-analyses should be included in future review studies. Second, although RT- and VR-MRCP-based BCI rehabilitation methods are promising neurorehabilitation methods for inducing plasticity in post-stroke patients, the generalization of the efficacy results is limited due to the small number of studies and subjects used in the review. Very few studies have conducted efficacy analyses of the tested methods in clinical settings. Therefore, we recommend that future studies evaluate the efficacy of RT- and VR-MRCP-based BCI rehabilitation methods using a large number of subjects in clinical settings.

## 5. Future Direction

Although we have highlighted the potential of RT and VR-MRCP-based BCI neurorehabilitation therapies for patients with motor impairment, high technological challenges make these new rehabilitation approaches challenging to use outside the controlled environment. Future improvements in hardware and software design and the reliability of neural signal acquisition methods, processing techniques, and signal classification methods should be carefully considered to encourage the use of these rehabilitation technologies in clinical settings elsewhere. To address these challenges, researchers with various professional backgrounds, such as clinical therapists, physicians, neuroscientists, robotic and software engineers, and others, must collaborate by disseminating technical skill information and pondering technological advancements to ensure the simplicity and reliability of the approaches. Furthermore, many subjects, both patient and healthy, should be included in the RT- and VR-MRCP-based BCI neurorehabilitation research. Therefore, this information could be beneficial in developing a patient-driven rehabilitation device based on MRCP-based BCI to induce brain plasticity and to restore motor impairment in the future.

## 6. Conclusions

The present systematic review focused on the RT- and VR-MRCP-based BCI neurorehabilitation techniques. The RT- and VR-MRCP-based BCI rehabilitation techniques are discussed in terms of their potential application, challenges pertaining to signal acquisitions, preprocessing, and processing methods. We also discussed the prospects intending to raise awareness of this emerging field. Adding these novel rehabilitation therapies to standard clinical therapies can improve the clinical success of motor impairment rehabilitation training by allowing for real-time functional assessment and effective treatments. The relationship between key items using the overlay visualization method is carried out, helping to identify the trend of the most frequent and recent key items used in this field. Due to the various challenges and limitations facing this field, as mentioned in Section 4 above, very few studies have been published from the searched databases. Perhaps, this is because of the newness and complexity of these rehabilitation techniques and the enormous demanding investment of cost and professionalism. Therefore, this study will support engineers and researchers in RT and VR- MRCP-based BCI systems to: improve clinical systems and methods, solve the current challenges, and enhance the usability of the RT- and VR-MRCP-based BCI rehabilitation systems for healthy persons and those with motor impairment.

## Figures and Tables

**Table 1:** Scores of methodological quality assessment of the included studies based on the PEDro scale.

Ref.	[49]	[15]	[50]	[51]	[52]	[53]	[54]	[55]	[56]	[47]	[57]	[58]	[59]	[60]	[61]	[62]	[63]	[64]
E	0	1	1	1	1	1	0	1	1	1	1	1	1	1	1	1	1	1
R	1	0	1	1	0	1	0	1	1	1	1	0	1	1	1	1	0	1
C	0	0	0	0	0	0	0	0	0	0	0	0	0	0	0	0	0	0
B	1	1	0	0	1	1	0	1	1	1	0	1	1	0	1	1	1	1
B^1^	0	0	0	0	0	0	0	0	0	0	0	0	0	0	0	0	0	0
B^2^	0	0	0	0	0	0	0	0	0	0	0	0	0	0	0	0	0	0
B^3^	0	1	0	0	0	0	0	0	0	0	0	0	0	0	0	0	0	0
M	1	1	1	1	1	1	1	1	1	1	1	1	1	1	1	0	1	1
I	1	1	1	0	0	0	1	1	1	1	1	1	1	1	1	0	1	1
B^4^	1	1	1	1	1	1	0	0	1	1	1	1	1	0	0	0	0	0
P	1	1	1	1	1	1	1	0	0	1	0	0	1	0	1	0	1	1
Total	6	7	6	5	5	6	3	5	4	6	5	5	7	4	6	3	5	6

E: Eligibility; R: Randomize allocation; C: Concealed Allocation; B: Baseline comparability; B^1^: Blinded subjects; B^2^: Blinded therapists; B^3^: Blinded assessors; M: Main outcomes; I: Intention -to- treat; B^4^: Between group statistical comparison; P: Point and variability measures.

**Table 2:** Applied rehabilitation methods in identified studies.

Studies	Rehabilitation Methods	Rehabilitation Tool Name
[15,52,62]	RT-MRCP-based BCI therapy	AMADEO
[51]		Lokomat
[54,56,57]		Rex,
[58]		X1 exoskeleton robot
[59]		BCI-MAFO
[47,50,58,63]	VR-MRCP-based BCI therapy	Controlling a virtual walking avatar and 2D cursor

**Table 3:** Research studies, electrode, and classifiers used, and the outcomes of RT- and VR-MRCP-based BCI neurorehabilitation techniques.

Ref.	Electrode	Epochs (s)	Bandpass Filter (Hz)	Classifier	Performance (%)
[47]	Fz, FC1, FC2, C3, Cz, C4, CP1, CP2, Pz	−2 to 0	0.04 to 3	MF-SVM	Sensitivity was 60.57 ± 14.79 for non-emergency tasks and 44.29 ± 5.73 for emergency tasks.
[49]	-	−2 to 1	0.1 to 2	MKL	Accuracy was above 90 for classifying gait states from EEG signals.
[52]	C3, FC3, CP3, Cz, T7	−2 to 0	0.1 to 1	SVM	Accuracy was 79.7 in healthy subjects and 66.64 in patients for movement execution trials.
[55]	Cz, Fz, FC1, FC2, C3, C4, CP1, CP2, Pz	−1 to 1	0.05 to 3	LPP-LDA	Sensitivity was 80 for the movement execution task and 70 for the intended task.
[56]	Cz, C1, C2, CPz	−2 to 1	0.1 to 4	RLDA	Accuracy was 87.6 for the walking intention task.
[57]	C1, C2, CPz, Cz	−2 to 1	0.05 to 2	RLDA	Accuracy was 86 in the generated dataset and 73 in the public dataset for the movement execution task.
[58]	Cz, CPz, FCz, C2, C1, CP1, CP2, C3	−1 to 2	0.1 to 1	LDA	Sensitivity was 83 for movement intention.
[59]	Cz, Fz, FC1, FC2, C3, C4, CP1, CP2, Pz	−1.5 to 0.5	0.05 to 3	LPP-LDA	Sensitivity was 73.0 ± 10.3 for movement intention.
[63]	Cz, Fz, FC1, FC2, C3, C4	−2 to 0	0.1 to 5	LPP-LDA	Accuracy was 97 for motor execution and 92 for motor imagery.
[64]	FCz, FC2, C1, Cz, C2, CP1, CPz, CP2	−1 to 1	0.1 to 1	SDA	Sensitivity was 84.44 in healthy subjects and 77.61 in patients for activating exoskeleton movement.

**Table 4:** Previously published papers evaluated based on subject, application, frequency, analysis, pace, and study description.

Ref.	Subj.	App.	Frequency	Analy.	Paced	Description
[15]	1 Patient	RT	3 blocks of 10 min every 3 days a week	Offline	Self	The use of EEG signals to improve the engagement of stroke patients using robot-assisted multisession rehabilitation training.
[47]	7 Healthy	VR	5 rounds, 5 min per round; 2–3 min of resting after two rounds	Offline	Self	MRCP detection concerning emergency and non-emergency tasks.
[49]	2 Healthy 1 Patient	RT	Multiple sessions in 30 days	Offline	Self	To compare the brain areas used for identifying movement intentions in healthy subjects and individuals with spinal cord injury.
[50]	2 Healthy	VR	Pre-exposure for 8 min, exposure for 15 min, and post-exposure for 8 min for 8 days	Online	Self	The use of the closed-loop BCI-VR technology to control the walking movements of a virtual avatar.
[51]	8 Patient	RT	Cumulated number of hours and sessions recorded after 4, 7, 10, and 12 months	Offline	Self	Long-term training with a BMI gait protocol induces partial neurological recovery in paraplegic patients.
[52]	4 Healthy 2 Patient	RT	6 blocks consisting of 23 trials	Offline	Self	Using an EEG-BCI system with robot-assistive technologies to improve the effectiveness of the hand motor skills in post-stroke patients.
[53]	21 Patient	RT	30–50 training trials of dorsiflexion of the foot	Offline	Self	The application of BCI to chronic Stroke led to an increased output of the motor cortex to the target muscle.
[54]	1 Patient	RT	8 trials followed by a 45-min break	Offline	Self	To decode the motion intentions of a paraplegic person and give him the ability to walk using a lower-body exoskeleton.
[55]	10 Healthy	RT	30 training trials	Online	Self	The capability of the user to discriminate between a set of external sensory stimuli combined with a fast and reliable BCI brain switch.
[56]	5 Healthy	RT.	50 trials, 9 s of one-step walking, and 10 s of resting	Offline	Self	Under the powered exoskeleton environment, decoding user intention.
[57]	10 Healthy	RT	50 trials of resting, walking intention, and exoskeleton walking	Offline	Self	To improve the performance of MRCP decoding.
[58]	6 healthy	VR	50 trials	Offline	Self	EEG activities were utilized to characterize the intention to move in rehabilitation procedures.
[59]	10 Healthy	RT	30 trials	Online	Self	MAFO is powered by a BCI for stroke rehabilitation, with evidence of its efficacy in promoting cortical neuroplasticity.
[60]	4 Healthy	RT	20 trials per run, with 5–8 s between each task	Online	Self	Neurofeedback study for variations in MRCP in real-time.
[61]	2 Patient	RT	5-min walk with robot-on, robot-off, and no-robot conditions	Offline	Cue	Implementation of multimodal physiological interface with the X1 device during walking.
[62]	3 Patient	RT	24 training sessions, each lasting for 30 min 3 days a week	Offline	Self	Application of two-stage robot-assisted training for hand motor recovery.
[63]	11 Healthy	VR	30 trials of ballistic dorsiflexion	Online	Self	Application of the new BCI to dynamic real-life scenarios: feasibility and benefit.
[64]	3 Healthy, 4 Patient	RT	For healthy subjects, 5–10 min of walking with the exoskeleton. For the patients, 20 and 30 min	Online	Self	Analyzing the indicators of the viability of the system for clinical purposes.

## Data Availability

The data used to support the study findings are available from the corresponding authors upon request.

## Supplementary Materials

The following supporting information can be downloaded at: https://www.mdpi.com/article/10.3390/bios12121134/s1, PRISMA checklist.

## Abbreviations

BCI: Brain–Computer Interface; EEG: Electroencephalography; MRCP: Movement-Related Cortical Potential; RT: Robot Therapy; VR: Virtual Reality; HBMIs: Hybrid Brain–Machine Interfaces; PRISMA: Preferred Reporting Items for Systematic Reviews and Meta-Analyses; MEG: Magnetoencephalography; NIRS: Near-Infrared Spectroscopy; fMRI: Functional Magnetic Resonance Imaging; SSVEP: Steady-State Visual Evoked Potentials; SMRs: Sensorimotor Rhythms; LFTD: Low-Frequency Time-Domain; PEDro: Physiotherapy Evidence Database; TPR: True Positive Rate; SSSF: Subject-Dependent and Section-Wise Spectral Filtering; DoF: Degree of Freedom; LPP: Locality Preserving Projection; LDA: Linear Discriminant Analysis; SDA: Sparse Discriminant Analysis; SVM: Support Vector Machine; MKL: Multiple Kernel Learning; RLDA: Regularized Linear Discriminant Analysis: LFDA: Local Fisher’s Discriminant Analysis; G.M.M.: Gaussian Mixture Model; MF: Matching Filter.
