# Supplementary material for: A Systematic Review of Virtual Reality and Robot Therapy as Recent Rehabilitation Technologies Using EEG-Brain–Computer Interface Based on Movement-Related Cortical Potentials
Source: Biosensors (Basel). 2022 Dec 6;12(12):1134. doi: 10.3390/bios12121134 (PMC9776155; doi:10.3390/bios12121134)
Supplement: Supplementary file 1 [file biosensors-12-01134-s001.zip › biosensors-1961712-supplementary.pdf]

PRISMA 2020 Checklist

Section and Topic	Item #	Checklist item	Location where item is reported
**TITLE**			
Title	1	Identify the report as a systematic review.	Title page
**ABSTRACT**			
Abstract	2	See the PRISMA 2020 for Abstracts checklist.	Title page
**INTRODUCTION**			
Rationale	3	Describe the rationale for the review in the context of existing knowledge.	Section 1
Objectives	4	Provide an explicit statement of the objective(s) or question(s) the review addresses.	Section 1
**METHODS**			
Eligibility criteria	5	Specify the inclusion and exclusion criteria for the review and how studies were grouped for the syntheses.	Section 2
Information sources	6	Specify all databases, registers, websites, organisations, reference lists and other sources searched or consulted to identify studies. Specify the date when each source was last searched or consulted.	Section 2
Search strategy	7	Present the full search strategies for all databases, registers and websites, including any filters and limits used.	Section 2
Selection process	8	Specify the methods used to decide whether a study met the inclusion criteria of the review, including how many reviewers screened each record and each report retrieved, whether they worked independently, and if applicable, details of automation tools used in the process.	Section 2
Data collection process	9	Specify the methods used to collect data from reports, including how many reviewers collected data from each report, whether they worked independently, any processes for obtaining or confirming data from study investigators, and if applicable, details of automation tools used in the process.	Section 2
Data items	7	List and define all outcomes for which data were sought. Specify whether all results that were compatible with each outcome domain in each study were sought (e.g. for all measures, time points, analyses), and if not, the methods used to decide which results to collect.	Section 2
Synthesis methods	8	Describe the processes used to decide which studies were eligible for each synthesis (e.g. tabulating the study intervention characteristics and comparing against the planned groups for each synthesis (item #5)).	Section 2
**RESULTS**			
Study selection	9	Describe the results of the search and selection process, from the number of records identified in the search to the number of studies included in the review, ideally using a flow diagram.	Figure 3
Study characteristics	10	Cite each included study and present its characteristics.	Section 3, Tables 3 & 4
**DISCUSSION**			
Discussion	11	Provide a general interpretation of the results in the context of other evidence.	Section 4
	12	Discuss any limitations of the evidence included in the review.	Section 4
	13	Discuss any limitations of the review processes used.	Section 4.1
	14	Discuss implications of the results for practice, policy, and future research.	Section 6
**OTHER INFORMATION**			
Support	15	Describe sources of financial or non-financial support for the review, and the role of the funders or sponsors in the review.	Section 10
Competing interests	16	Declare any competing interests of review authors.	Section 11

PRISMA 2020 Checklist

Section and Topic	Item #	Checklist item	Location where item is reported
Availability of data, code and other materials	17	Report which of the following are publicly available and where they can be found: template data collection forms; data extracted from included studies; data used for all analyses; analytic code; any other materials used in the review.	Section 12

From: Page MJ, McKenzie JE, Bossuyt PM, Boutron I, Hoffmann TC, Mulrow CD, et al. The PRISMA 2020 statement: an updated guideline for reporting systematic reviews. BMJ 2021;372:n71. doi: 10.1136/bmj.n71

For more information, visit: http://www.prisma-statement.org/
